# Overview of the taxonomy of zooxanthellate Scleractinia

**DOI:** 10.1111/zoj.12076

**Published:** 2013-10-11

**Authors:** John Veron

**Affiliations:** Coral Reef Research10 Benalla Road, Oak Valley, Townsville, QLD, 4811, Australia; University of QueenslandBrisbane, QLD, 4064, Australia; James Cook UniversityTownsville, QLD, 4811, Australia

**Keywords:** biogeography, coral, historical taxonomy, molecular taxonomy, morphological taxonomy, Scleractinia, taxonomy

## Abstract

Coral taxonomy has entered a historical phase where nomenclatorial uncertainty is rapidly increasing. The fundamental cause is mandatory adherence to historical monographs that lack essential information of all sorts, and also to type specimens, if they exist at all, that are commonly unrecognizable fragments or are uncharacteristic of the species they are believed to represent. Historical problems, including incorrect subsequent type species designations, also create uncertainty for many well-established genera. The advent of *in situ* studies in the 1970s revealed these issues; now molecular technology is again changing the taxonomic landscape. The competing methodologies involved must be seen in context if they are to avoid becoming an additional basis for continuing nomenclatorial instability. To prevent this happening, the International Commission on Zoological Nomenclature (ICZN) will need to focus on rules that consolidate well-established nomenclature and allow for the designation of new type specimens that are unambiguous, and which include both skeletal material and soft tissue for molecular study. Taxonomic and biogeographic findings have now become linked, with molecular methodologies providing the capacity to re-visit past taxonomic decisions, and to extend both taxonomy and biogeography into the realm of evolutionary theory. It is proposed that most species will ultimately be seen as operational taxonomic units that are human rather than natural constructs, which in consequence will always have fuzzy morphological, genetic, and distribution boundaries. The pathway ahead calls for the integration of morphological and molecular taxonomies, and for website delivery of information that crosses current discipline boundaries.

As most users of coral taxonomy appreciate, this notoriously subjective science has gone through three historical phases: (1) studies of collections made during early expeditions of discovery; (2) reef-based studies using scuba; and (3) molecular studies. These phases are each linked to such different methodologies and perceptions that they have little in common; however, they do have a common goal, which is to classify corals according to a concept of natural order. To elucidate this history, two of the world's best-known species *Pocillopora damicornis* (Linnaeus, 1758) and *Porites lobata* Dana, 1846 are used as examples.

## The Tyranny of the Past

Corals, more than any other group of marine invertebrates, with the possible exception of molluscs, were the most sought-after undersea collectables of early expeditions of discovery to the tropical world. This was because of the close association of corals with coral reefs, considered then as now to be amongst the most exotic natural wonders on earth. It was also because corals could be easily collected in large quantities and stowed in the holds of ships without need of further care. Furthermore, corals made excellent museum exhibits, especially when painted gaudy colours to supposedly resemble their living appearance.

### Historic collections

With few exceptions, corals were collected or purchased because individual specimens appeared to be new or unusual, rather than because they were representative of a population or a taxonomic group. They were also collected from shallow habitats such as reef flats, places where branching and plate-like colonies usually develop unusual growth forms. These collections thus introduced a sampling bias that has plagued taxonomic studies ever since, and resulted in a proliferation of type specimens that do not clearly represent the species they are intended to define.

By such means, for over 200 years, corals were accumulated in great quantity in museums across Europe and the USA, collections considered valuable contributions to Natural History, especially when made the subject of monographs, of which a great many were published. In historical perspective these publications were usually works of art as much as science, seen for example in the unsurpassed artwork of Müller (1775), Ellis & Solander (1786), Stutchbury (1830), de Blainville (1834), Michelin (1840), Milne Edwards & Haime (1848, 1850), Dana (1846), Haime & Milne Edwards (1857), Duchassaing & Michelotti (1860), Agassiz (1880), or Haeckel (1876), where authors sought to impress a wider scientific community as much as document the taxonomic characters of corals. Thus, despite their status as being among the most scholarly monographs of their time, the species descriptions they contain are usually unhelpful, for they lack details of morphology (especially about how one species might be distinguished from another), habitat, and even location. For this reason, modern taxonomists must rely on type specimens and illustrations rather than descriptions to determine the actual identity of the species being described.

In recent years, historic collections, and the studies made of them, have become the bane of coral taxonomy, for they tie modern studies to an antiquated past via rules of nomenclature that may have little intrinsic value, and instead have an endless capacity to maintain uncertainty, even where, as far as the actual corals are concerned, there is none.

### Type specimens

Coral taxonomists of the remote past were not divers, and therefore had no idea how species actually appeared in nature, including variation in their shape, colour, and abundance. If a specimen looked different enough it was proclaimed a new species and given a name; there was no concept of what species actually were. At this time also, corals were swapped or borrowed among naturalists or museums for the price of a postage stamp, perhaps to be returned later, perhaps not. Inevitably many specimens were lost or now appear to be lost because they were given a new label and incorporated into another collection, commonly without any indication of their original source. With some exceptions, type specimens were not considered as essential as they are today, nor were different categories of types recognized. Many remained unmarked, later to be revealed as a type specimen on the basis of the handwriting on their label or a particular form of notation used by an individual author. It is also likely that many type specimens now believed lost never existed, as they were no more than interesting specimens selected for illustration and description, then returned to a general collection once that job was done.

Although historic type specimens are given equal status today, some are deserving of a special status whereas others are not. For example, James Dana, the most astute coral taxonomist of the 19^th^ century, was particularly precise about his specimens and accurate in his descriptions, but not so A. E. Verrill who, following in Dana's footsteps, designated type specimens from barely recognizable fragments, which he deposited in different museums (Verrill, 1864). Some of Verrill's types found in the Museum of Comparative Zoology, Harvard University, and the Smithsonian Institution today were clearly taken from different colonies that actually belong to different species, with some bearing a reference to Dana, and others not. A more general problem was the casual treatment of type specimens by some museums. Many types have been supposedly lost, then found, or declared to be types when they are not. For example, in the mid-1970s the Paris Museum proudly displayed the historic type specimens of Lamarck and his contemporaries, but not those of subsequent authors, notably Milne Edwards and Haime, many of which were kept in general collections. The present author, helped when time permitted by the museum's coral palaeontologist, Jean-Pierre Chevalier, attached explanatory notes to what were probably some of Milne Edwards' and Haime's type specimens that had been presumed lost. This is not just a matter of historical anecdote, nor confined to the Paris Museum: curation of type specimens directly affect today's taxonomic decisions, and helps to ensure that problems of the past are kept alive and continue to destabilize species nomenclature. This issue is further pursued below.

### Type species

The value of type species, the species on which genera are based, seems obvious; however, in practice, using type species as a basis for taxonomic decisions is seldom a useful process because they are usually among the first-described species of a genus, and represent a distillation of the sorts of historical problems just described. Any coral taxonomist choosing to update the type species documented by Vaughan & Wells (1943) and Wells (1956) is in for surprises. For example, the type species of genus *Leptoseris* is *Leptoseris fragilis* Milne Edwards & Haime, 1849, about which so little is known that it was not re-described in Dinesen's (1980) revision of that genus, nor included in Veron (2000a). At least *L. fragilis* is almost certainly a *Leptoseris*, but the identity of the type species of other genera is less certain. For example, the type species of *Montastraea* is *Astrea guettardi* de Blainville, 1830, a long-lost Miocene fossil, debatably from France or Italy, that is unidentifiable.

Such cases have been ignored in accordance with the old adage ‘let sleeping dogs lie’, but this can leave genera prone to a takeover. For example, the type species of *Favia* Oken, 1815 is supposedly the Atlantic species *Favia fragum* (Esper, 1797), designated the type species by Verrill (1901). This is another example of Verrill's propensity for mistakes, as *F. fragum* is unlikely to be a *Favia* at all, although that is what it has always been called because of its type-species status. It is widely believed that the only solution to such a historical error is to give all other *Favia* species (except the close ally of *F. fragum*, *Favia gravida* Verrill, 1868) a different generic name, as Budd *et al*. (2012) have recently done (see ‘Ockham's razor’ below). In fact, all Oken's genera (including *Favia*, but also *Acropora*, *Galaxea*, *Mussa*, *Mycedium*, *Pectinia*, and *Turbinaria*) are technically invalid or ‘unavailable’ in the language of the International Commission on Zoological Nomenclature (ICZN Commission, 1956), unless rescued by subsequent designations because Oken did not adhere to binomial nomenclature. In the particular case of *Favia* one may well ask: should an obscure 200-year-old publication, supposedly corrected by a 100-year-old mistake, matter when the name *Favia* has now been used unambiguously in over a thousand publications? Obviously not, especially as subsequent designations are matters of opinion, which may not accord with the views of other taxonomists, or indeed with those of the original author of the genus.

In total, and irrespective of the rules, 20 genera (*Astrangia*, *Balanophyllia*, *Colpophyllia*, *Coscinaraea*, *Diploria*, *Goniopora*, *Leptoria*, *Leptoseris*, *Meandrina*, *Montastraea*, *Oculina*, *Pavona*, *Podabacia*, *Polyphyllia*, *Porites*, *Seriatopora*, *Solenastrea*, *Stephanocoenia*, *Trachyphyllia*, and *Turbinaria*) have unrecognizable type species, and as it currently stands the validity of all these names lack certainty for one historical reason or another. Nevertheless, the stability of generic names has been perfectly adequate without type species and the baggage that goes with them. Even *Acropora*, the best known of all coral genera, was only validated by the ICZN in 1963.

In fact, despite common beliefs, the ICZN offers alternatives to name-changing, including ratification of existing names, where older names take priority, and the designation of new type species.

Type genera of families have even less relevance to the real world, and are generally subsequent designations ignored by most authors.

### International commission on zoological nomenclature

Today we are left with a taxonomic legacy from the past that has more to do with human history than taxonomy. This is particularly unfortunate in the case of corals but it is far from unique to them. In order to put zoological nomenclature into some semblance of order, the ICZN (which produces and periodically updates the *International Code of Zoological Nomenclature*) was founded in 1895 and, funded by a charitable trust, has since done much to tidy-up general taxonomic problems as well as specific details relevant to individual publications or taxa.

The original premise of the ICZN was that taxonomic decisions should reduce uncertainty, not increase it, a critical goal that is now often overlooked by taxonomists, and sometimes even by the ICZN itself. As a paralegal organization it is fitting that the ICZN should be concerned with regulation; however, it is critical that its membership maintains focus on the real needs of a rapidly changing taxonomic – and technological – landscape. For example, following years of ‘highly charged debate’ the ICZN has only recently allowed descriptions of new taxa to be published electronically, and even today there are basic issues concerning the use of Latin. This was once a language firmly entrenched in the international law, religion, history, astronomy, anatomy, and taxonomy of the western world, but it is no longer, and yet the ICZN still requires that the rules of Latin declension take priority over names that an unwary taxonomist might create, even to the point that a species name must be changed to match the gender of its genus should this be changed. This is a guaranteed recipe for disorder in an age of electronic information searches. The simple alternative, placing the needs of stability and information technology above that of Latin grammar, would simply be to retain original spellings.

As far as corals are concerned, the emergence of molecular taxonomy based on living tissue and not skeletons renders all but some of the most recent holotypes (that have living tissue preserved) inadequate for future taxonomic and biogeographic studies (see ‘Where molecular taxonomy and biogeography meet’ below). Regulatory changes to address this issue need to be in place, the principle being that some faunal groups have specific nomenclatorial requirements that do not arise elsewhere.

Equally important is the need for the ICZN to put an end to the name-games commonly being played with corals, as these are relicts of history and have nothing to do with the corals themselves.

### Name-games

Because of historical inheritance, even the best-known species names are vulnerable to change because of the widespread belief that the oldest name must be the one accepted, despite the fact that these are often the least certain. For example, Wallace (1999) changed the name *Acropora formosa* (Dana, 1846), probably the most widely and reliably cited of all *Acropora* species, to *Acropora muricata* (Linnaeus, 1758) on the basis of one doubtful drawing (which could be one of several staghorn *Acroporas*), evoking nomenclatorial priority as the reason for doing so. In this case the motive was presumably to provide a neotype for *A. muricata*, the type species of *Acropora*; however, another problem that has greater potential to proliferate is the creation of a new name because of the perceived misidentification of an old type specimen. For example, *Madracis mirabilis* (Lyman, 1859), another widely known and reliably cited species, was renamed *Madracis auretenra* by Locke, Weil & Coates (2007) in order to sort out a problem they believe occurred with type specimens. Such views are seldom straightforward and many are unique to individual species, but in the interests of keeping scientific publications relevant and understandable for non-taxonomists, there would again need to be very good reason to change a commonly used name that is unambiguous. The designation of a new holotype where needed would be a simple way of retaining such names.

Some recent authors have retained names in current use by suppressing older names. For example, Benzoni *et al*. (2010) retained the name *Psammocora nierstraszi* Van der Horst, 1921, although she found that *Psammocora verrilli* Vaughan, 1907 had priority, a procedure allowed by ICZN article 23.9.3. Nomenclatorial stability would be well served if this process was explicitly recommended or mandatory, rather than discretionary.

It is tempting to believe that these sorts of issues will eventually sort themselves out; however, this is unlikely. Of the estimated 2400 nominal extant zooxanthellate coral species in existence, 15% have no taxonomic records and those that do have taxonomic records have their names imbedded in the vagaries of nomenclatorial history. This leaves a large number of species exposed to name changes on the grounds of nomenclatorial priority. In many cases there are good reasons for changing names, corrections of mistakes being the main one; however, some recent authors have not considered stability, and seem to be unaware of any need to do so.

In summary, it is hard to avoid the conclusion that, unless remedies are found, name-games that reduce certainty will remain a permanent fixture of coral taxonomy, yet this would not be so if established names were retained when their identity is clear, and when new type specimens (with soft tissue preserved) are used to augment, or replace, old holotypes. There are many procedural problems with such a process; however, something like it will eventually become necessary if coral taxonomy is to avoid an unending decline in stability. Perhaps the finding of a solution will be a future task of the ICZN, advised by members recruited from the ranks of molecular biology. This subject is continued below after brief consideration of the different methodologies used in fossil and extant coral taxonomy.

### Fossils, taphonomy, and microcrystalline structure

There is enormous intrinsic interest in the evolutionary history of corals, for corals are nature's historians, revealing more about Mesozoic and Cenozoic marine environments than any other faunal group (Veron, 2008). For these reasons the palaeontological literature, particularly that dealing with Mesozoic corals, is extensive. The international repository, Paleobiology Database, offers a wide range of theoretical nominal taxa, including over 6000 species of Scleractinia. These do not have anything like the taxonomic reliability of extant coral species, but at the generic level the database is highly informative. Nevertheless there are limits: our detailed knowledge of Palaeozoic corals (Rugosa and Tabulata), which have skeletons of calcite, is not mirrored by the Scleractinia, which have skeletons of aragonite. The processes of diagenesis, where the original aragonite of scleractinian skeletons is replaced by calcite, destroys skeletal detail, thereby limiting our knowledge of skeletal structures of fossils to discoveries where at least some surface structure and/or macro-morphology has been preserved. The alternative is to use the technique of thin sectioning of specimens in which at least some aragonite has been retained or where diagenesis has been relatively benign (reviewed by Stolarski & Roniewicz, 2001). However, the nomenclature and proposed relationships between extinct families based on such studies (see ‘Family trees’ below) is the subject of often fundamental disagreement, stemming from a reliance on techniques used to try to overcome information loss through fossilization (taphonomy) and alternative interpretations of the identity of individual specimens or groups of specimens.

Thin sections and etching can also be used to study the microcrystalline structure of extant corals, especially applicable to families that have distinctive wall, horizontal, or septal elements. However, skeletal microstructure (documented by Stolarski, 2003 in fossils) has yet to be investigated in living corals in anything like the detail needed to underpin a taxonomic hierarchy, and indeed microstructure was not fundamental to the (primarily fossil) compendiums of Vaughan & Wells (1943), Wells (1956), or Chevalier & Beauvais (1987), see ‘Ockham's razor’ below.

Today, environment-correlated microskeletal variation remains unstudied, even at the generic level, yet this variation is readily seen in most faviid and mussid species. For example, *Lobophyllia pachysepta* Chevalier, 1975 and *Symphyllia agaricia* Milne Edwards & Haime, 1849 both have thick, granulated, club-shaped tips to their septal dentations in wave-hammered environments, grading to thin smooth pointed dentations in protected environments. Likewise, the wall structure of *Oulophyllia crispa* (Lamarck, 1816), for example, changes from being primarily septothecal to being primarily parathecal with decreasing exposure to wave action. Furthermore, the ontogeny of skeletal elements has yet to be documented in taxonomic detail, despite the fact that growth from early postlarval stages (‘spat’) to adult colonies is routinely observed on settlement plates.

For good reasons, the names of fossils have rarely been applied to extant corals. Two of the three species of the *Montastraea annularis* group used in the molecular studies of Knowlton, Budd, and their associates (originally in Weil & Knowlton, 1994) are exceptions. Of these, *Montastraea faveolata* (Ellis & Solander, 1786) has a fossil holotype that has been so changed by diagenesis that it cannot reasonably be ascribed to a genus, let alone a species. Thus, for this well-studied coral, neither genus nor species are based on recognizable type specimens. In principle, fossil type specimens, and the names that go with them, should be avoided for extant corals, or at least have type specimens of extant corals nominated for inclusion with them.

## The Reality of the Reef

Observing corals in their natural environment using scuba became a tool – virtually a way of life – used by coral taxonomists from the early 1970s. At that time there were three schools that spanned the whole taxonomic spectrum. An American school of geologists, stemming from James Dana and progressing through T. W. Vaughan to John Wells, was the primary taxonomic information source of the time. There was also the Japanese school of Yabe, Sugiyama, and Eguchi, less well known but productive, which ended for the most part after the Second World War. Finally, there was the Philippines school of Faustino (1927), followed by the many publications of Francisco Nemenzo and his associates, which were still current when *in situ* studies had become popular. All embraced the same taxonomic history described above, and all relied on the same principal monographs, especially the seven volumes of the *Catalogue of the Madreporian Corals in the British Museum (Natural History)* (Brook, 1893; Bernard, 1896, 1897, 1903, 1905, 1906; Matthai, 1928) and a succession of Dutch publications, notably from the Rijksmuseum. Despite the use of the same historical information sources, the taxonomies of these three schools had little in common except for the names they used, names that were commonly applied to completely different species. The reasons for this appear to be: (1) an almost total lack of information exchange between contemporaneous authors, perhaps because this was deemed to be a matter best dealt with in synonymies; (2) the Japanese and Philippines schools tended to be insular in the face of the Americans who had the resources for foreign travel; and (3) the study of type specimens in foreign museums was not accorded the importance that it now has.

Wells' (1954) *Recent corals of the Marshall Islands* was widely considered the most authoritative work of the pre-scuba era, and underpins most taxonomic studies from then until the early 1970s.

### Species *in situ*

**Figure 1:**
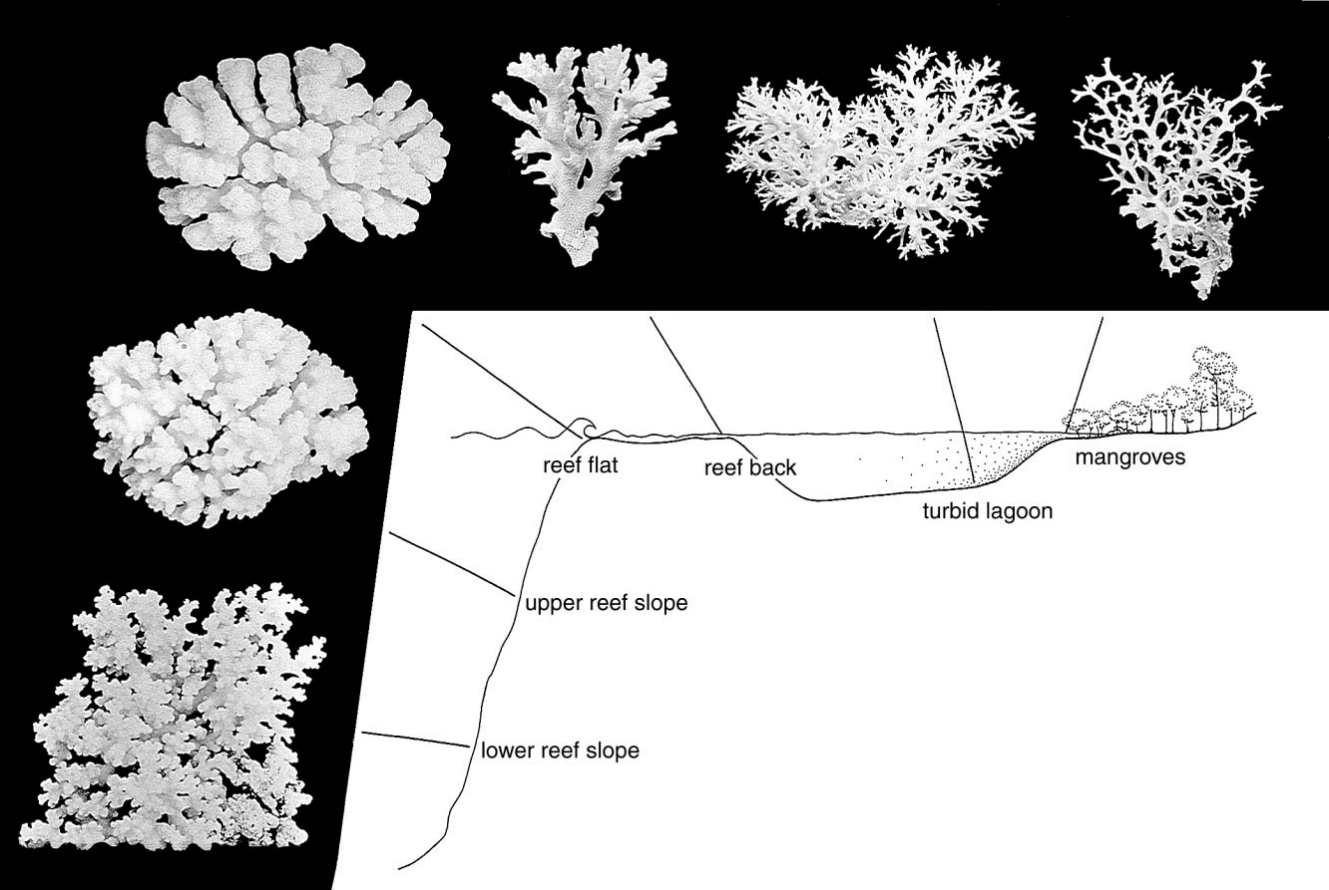
Variation of the skeletal structure of *Pocillopora damicornis* from a wide range of habitats, as illustrated by Veron and Pichon (82).

**Figure 2:**
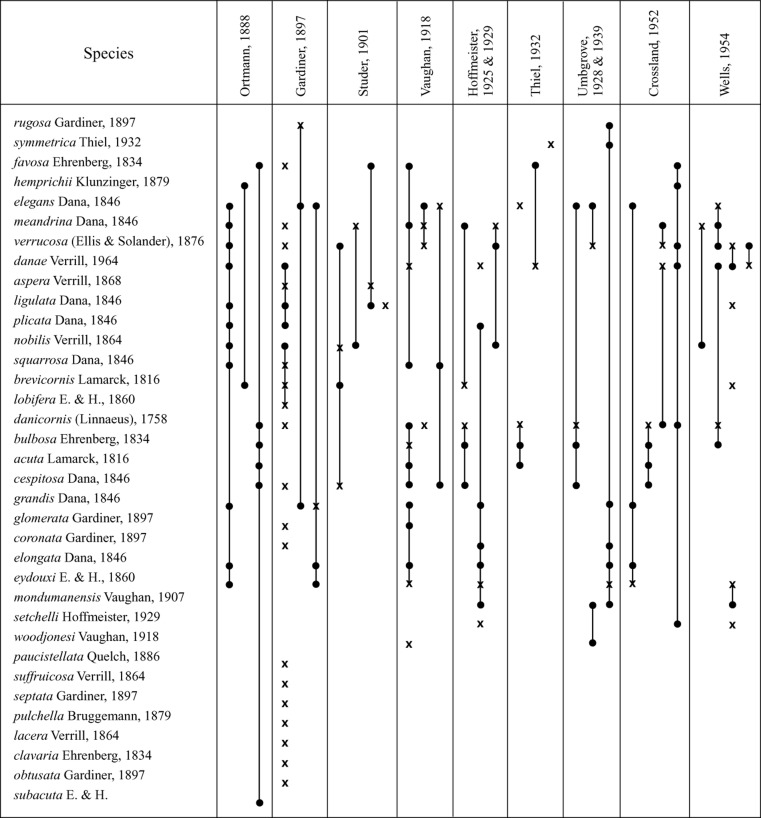
Species of *Pocillopora* and their synonyms, as illustrated by Veron & Pichon (82). ×, species considered valid; •, synonyms, connected by a vertical line.

Taxonomic studies using scuba commenced in the early 1970s and immediately created major conflicts with virtually all aspects of the traditional taxonomy of the time. For example, *Pocillopora damicornis*, recorded in over 50 taxonomic publications, and about twice that number of non-taxonomic research papers before 1970, was the most commonly used species of experimental research. The questions that naturally arose were: (1) what actually is *Pocillopora damicornis*; (2) how could it be reliably distinguished from other *Pocillopora* species; and (3) what is its distribution? Linnaeus' original description (in just 20 words of Latin) is not remotely helpful, the holotype is lost, and the type locality is impossibly vague (‘O. Africano & Indico’). More importantly, when (the presumed) *Pocillopora damicornis* was seen *in situ* it showed so much environment-correlated variation (Fig. 1) that the taxonomic accounts of the time left almost every issue unanswered. The second question posed worse problems, for a perusal of the taxonomic literature of *Pocillopora* revealed little else but disagreement (Fig. 2).

*In situ* studies of corals offered many solutions: (1) they allowed species to be identified with much greater reliability; (2) they allowed comprehensive surveys to be undertaken; (3) they provided distinct criteria by which closely related species could be distinguished; and (4) they revealed how changes in skeletal morphology are correlated with the environment. This work reinvigorated coral taxonomy and led to detailed studies of at least one million specimens worldwide. Forty years on, *in situ* studies are still evolving, providing a solid foundation for a wide range of research as well as overwhelming support for reef conservation.

### The taxonomic hierarchy

Taxonomic hierarchies are the outcome of grouping species into genera and genera into families. Although it seems a statement of the obvious, it is only possible to build a hierarchy from the bottom up: genera must be founded on species and families on genera if the hierarchy is to accurately reflect what occurs in nature. A perfect taxonomic hierarchy of Scleractinia would be based on: (1) all species being taxonomically isolated units; and (2) every species included. In the real world these conditions can never be met.

### Categories of species

Species that are sufficiently well known to be currently used as operational taxonomic units (Veron, 2000a, and subsequent additions) can be attributed to one of the following categories, according to their taxonomic history.

#### Very distinct species

Monospecific genera all have very distinctive species (Veron 2000a and subsequent additions).

#### Well-defined species

Despite their reputation for being taxonomically difficult, the majority of species that belong to most major genera can be reliably identified within a geographic region because they have one or more conspicuous characters that display little variation.

#### Apparently well-defined species

Many species appear to be taxonomically straightforward, but are so variable that appearances might be deceptive. For example, *Pavona maldivensis* (Gardiner, 1905) has such distinctive characters that it is readily recognized over its Indo-Pacific-wide distribution range; however, because it exhibits wide variation in most skeletal characters, this conclusion awaits confirmation by molecular study.

#### Problematic species

Most of these species can be reliably identified by an expert in a particular region, but less reliably over a wide geographic range. For example, *Pocillopora damicornis* can usually be identified with a high level of certainty in the central Indo-Pacific, but this is progressively more problematic in more distant regions. This is not a lack of expertise on the part of the taxonomist, it is the outcome of reticulate pattern formation (see ‘The Last Frontier’ below).

#### Species complexes

Many species might appear to be taxonomically straightforward but are not. For example, *Montastraea annularis* (Ellis & Solander, 1786), once considered a single species, was found to be a complex of three species, as mentioned above, by molecular studies (Knowlton *et al*., 1992; Weil & Knowlton, 1994). These could have (and should have) been recognized from *in situ* morphological studies, but were not. Many other old and well-known species, for example *Cyphastrea serailia* (Forskål, 1775) and *Lobophyllia hemprichii* (Ehrenberg, 1834) are almost certainly species complexes of a similar kind, yet have not been subdivided despite determined attempts to do so. For this reason they have extensive synonymies (Veron, Pichon & Wijsman-Best, 1977 and Veron & Pichon, 1976, respectively) that await molecular confirmation.

#### Species based on a **lack** of characters

**Figure 3:**
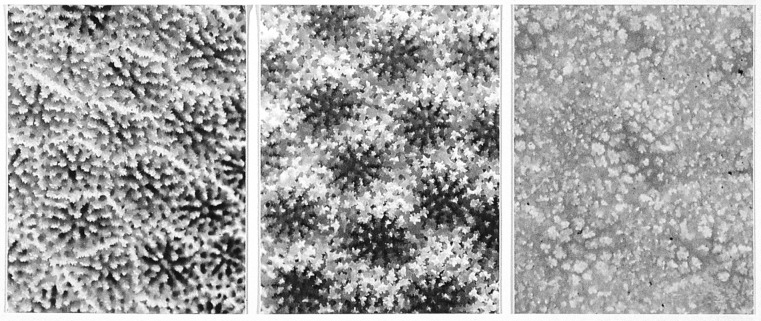
Corallite variation within a single colony of *Porites lutea*. The corallites occur within 300 mm of each other, around the lip of the base of a helmet-shaped colony. After Veron (1995).

This seemingly obtuse concept is probably commonplace in a few genera. For example, massive *Porites* species are identified on the basis of calice characters that may vary so much that different corallites of the same colony have few if any structural elements in common (Fig. 3). This can partly be controlled for when identifying *Porites* species; however, the concept that some species are single entities with an Indo-Pacific-wide distribution is primarily based on details of septa, characters that may not be adequate for such a purpose. *In situ* studies have established reliable criteria for separating *Porites* species where they co-occur; however, such distinctions over wide geographic ranges await confirmation using molecular methods. These are likely to reveal an array of cryptic species, for example *Porites paschalensis* Vaughan, 1906 from remote Easter Island is usually considered a junior synonym of *Porites lobata* Dana, 1846, which spans the entire Indo-Pacific. Whether this is so or not is beyond any morphological study to determine because *Porites* does not have adequate morphological characters to support such studies.

### Subspecies taxon levels

It was once commonplace for variations in a well-studied species to be called ‘forma’, ‘varieties’, or ‘subspecies’, and given individual names in the belief that these were distinct taxa. For example, Vaughan (1907), in 11 pages of detailed description, divided *Porites lobata* in Hawaii into ten named ‘forma’ and ‘subforma’; however, if Vaughan could have studied large colonies *in situ* he would have found that different parts of the same colony commonly exhibit the characters of several of his forma. In principle, subspecies taxon levels are artificial groupings, although many coral species, as with plants, have local or even widespread populations that have distinctive colours as well as minor morphological characteristics.

Cladograms, which indicate ever more divisions, are likely to lead to a future revival of subspecies taxon levels. These are of doubtful value in the context of ever-changing morphological and geographic continua.

Most species exhibit environment-correlated variations, the individual components of which may usefully be termed ‘ecomorphs’. Ecomorphs are not a taxon level because they are arbitrary and merge with each other within the same species (as with *Pocillopora damicornis*, Fig. 1). Significantly, the formation of ecomorphs may not entirely result from environment-correlated plasticity in growth form, as there may be significant selection of specific genotypes in colonies growing in stressful or otherwise marginal environments.

There is often an unclear distinction between an ecomorph and a species. For example, colonies exposed to wave action in Figure 1 might be an ecomorph of *Pocillopora damicornis* or almost equally the separate species *Pocillopora brevicornis* Lamarck, 1816, depending on morphological details revealed by molecular studies (see ‘Molecular taxonomic tools’ below).

### Geographic limitations

Clearly, studies of intraspecific variation along environmental gradients can be made in any geographic region where the species occurs; however, morphological studies to reveal how closely related species differ are obviously restricted to regions where they co-occur, usually places where diversity is high. For this reason, isolated locations have a high proportion of unresolved taxonomic problems at species level that can only be studied by molecular methods. For example, Veron *et al*. (1974) assumed that the *Pocillopora* specimens occurring at the Solitary Islands of high-latitude eastern Australia were an aberrant form of *Pocillopora damicornis* restricted to the extreme environment of this location, whereas molecular methods have shown this to be a distinct species, *Pocillopora aliciae* Schmidt-Roach, Miller & Andreakis, 2013a.

In principle, studies of Indo-Pacific corals based on morphology are at their most reliable in regions of high diversity (where a species and its close allies are most likely to co-occur), and are least reliable in remote regions (where they are unlikely to co-occur). In contrast, Caribbean species have a high level of uniformity in both occurrence and variability. Thus, results of taxonomic studies in one Caribbean country are generally applicable to other countries within the region.

### Morphometrics, cladistics, and pattern recognition

The analysis of measurements of corallite skeletal structures – morphometrics – has been used to support taxonomic observations since the 1980s (Willis, 1985). The main attractions of the method are in the name (which suggests objectivity), numerical rigour, and repeatability.

Cladistic or principal component analysis can greatly enhance the value of morphometric data, provided that clade generation is not used to extend clade distinctions to levels beyond the information value of the original data. In practice, there is an invisible line between cladistics used for data sorting and cladistics used for numerical taxonomy (Sokal & Sneath, 1968). The former is the preferred tool of data analysis today, whereas the latter is generally considered a tool of last resort stemming from a time when species were believed to be reproductively isolated units (reviewed by Veron, 1995), and even Willi Hennig himself (Hennig, 1966) warned that cladograms can create false divisions where there has been hybridization between the taxa under study (see ‘The last frontier’ below).

As far as corals are concerned, morphometrics does not reveal differences between corallites that are not readily seen by skilled observers (with humans being particularly adept at pattern recognition), and the methodology has severe limitations. For example, old corallites near the base of mature *Pocillopora damicornis* colonies usually have more in common with basal corallites of other *Pocillopora* species than they have with peripheral corallites of their own colony. This is readily seen at a glance; however, morphometrics can no more accommodate it than it can meaningfully accommodate variation in corallite morphologies among colonies from different environments. In theory this level of variation can be controlled for by selecting corallites according to specified criteria (such as distance from a branch tip); however, this brings into question the value of using morphometrics in the first place. Massive *Porites* colonies illustrate a similar issue: corallites from the basal parts of helmet-shaped colonies may have almost nothing in common with those on the upper surface (Fig. 3), something readily observable but not readily captured by morphometrics. In general, morphometrics is only of value in specific circumstances, and even then is of dubious value as a stand-alone basis for taxonomic decisions.

Pattern recognition computer technology (as used in facial or fingerprint recognition) is another matter, for such programs have the potential to rapidly record unlimited detail in corallite variation. So far, pilot studies on skeletons rather than living colonies have not been published, but presumably the methodology has enormous potential to link morphology and molecular data.

### Field guides

Field guides and electronic keys that illustrate species and distinguish between them *in situ* for particular regions are an effective intermediary between the complexities of taxonomy and the needs of non-taxonomists to identify corals. They are also a valuable illustration of the reality of reef studies and of broad-scale geographic distributions. Approximately twenty species-level field guides to corals have been published that usefully illustrate the key identifying characters of living colonies in the region they cover. Like field guides to birds, they are designed to illustrate characters used in recognition, and usually do so more effectively than taxonomic publications.

### Genera and binomial nomenclature

Most of the common genera of corals are well defined to the point of being obvious; however, some are not. The concept of binomial nomenclature requires that all species must be assigned to a genus, irrespective of whether this is a clear decision or a best guess. This sometimes forces taxonomists to designate a genus when the identity of the species is clear but the genus is not.

### Categories of genera

Just as all species are not taxonomically equal, genera based on morphological characters as opposed to molecular characters (see ‘Molecular taxonomic tools’ below) can be attributed to one or more of the following categories. Note that a genus may be well defined even when it contains doubtful species and *vice versa*.

#### Well-defined genera

Most coral species can be attributed to a genus with a high degree of certainty and with minimal taxonomic expertise. Of the 114 genera recognized by Veron (2000a) with subsequent additions, 85 belong to this category.

#### Well-defined genera with exceptions

Some genera are mostly well defined but contain uncertain species, with the uncertainties having multiple origins. These uncertainties can be resolved, left as problems to await further study, or given a new generic designation. For example, *Echinomorpha nishihirai* (Veron, 1990), *Australogyra zelli* (Veron *et al*., 1977), and *Australomussa rowleyensis* (Veron, 1985) were all removed from their original genus following further study.

#### Genera with uncertain boundaries

The boundaries of some large genera are linked to their taxonomic history, not because of an adherence to the past but because of a want of good reasons to make changes. For example, *Favia* and *Favites* would be well-defined genera were it not for some species that have almost equal affiliation to both, a problem exacerbated by the fact that environment-correlated variation within these species (notably a tendency to have common walls in high-energy environments and separate walls in protected environments) span both genera (Veron *et al*., 1977). This problem is particularly common among faviids.

#### Genera of convenience

Some species are attributed to genera that are essentially artificial because of the requirement of binomial nomenclature. Throughout the history of coral taxonomy, genera have been used or discarded on points of technicality, which may or may not have a phylogenetic basis. For example, the two species included in *Barabattoia*, *Barabattoia amicorum* (Milne Edwards & Haime, 1848) and *Barabattoia laddi* (Wells, 1954), have skeletal characters that exclude them from both *Favia* and *Montastraea*. In a similar vein, *Plesiastrea devantieri* Veron, 2000 and *Leptoseris yabei* (Pillai & Scheer, 1976) are well-defined species but do not clearly belong to the genus assigned them. Many such species await further study using molecular methods.

#### Alternative genera

Most instances where alternative generic names are commonly used are the result of revisions of earlier decisions, for example the separation of *Isopora* from *Acropora* by Wallace *et al*. (2007). Other alternative generic designations are sometimes used because they are recent changes to well-established genera that users may not be aware of. For example, *Galaxea horrescens* (Dana, 1846) was a monospecific species of *Acrhelia* until newly discovered species clearly linked these genera together. Alternative generic designations are also used for the Caribbean species *Leptoseris* (= *Helioseris*) *cucullata* (Ellis & Solander, 1786) and *Isophyllia* (= *Isophyllastrea*) *rigida* (Dana, 1846), the first because of similarities with Indo-Pacific *Leptoseris*, the second because of similarities with *Isophyllia sinuosa* (Ellis & Solander, 1786) (Veron, 2000a). These are again matters of opinion until molecular studies confirm one way or the other. Similarly, solitary fungiids are commonly given alternative designations because of continuing changes to the status of *Cycloseris*, *Fungia*, subgenera of *Fungia*, and *Diaseris*, partly reflecting the different treatments of these genera by Veron & Pichon (1976), Hoeksema (1989), and Veron (2000a), and partly a sequence of changes stemming from molecular studies, most recently by Gittenberger, Reijnen & Hoeksema (2011).

Subgenera, once widely used for *Fungia*, *Porites*, and some minor genera, are now out of use; however, they are likely to be revived to reflect the detailed resolution of the species clades generated by molecular data.

### Families

The family taxon level has not had the same level of interest in extant coral taxonomy as it has for fossils, where families are more in contention and there are more of them. Nevertheless, molecular studies will change many family divisions based on morphology and greatly increase the total number of families accepted.

### Categories of families

Families, like genera and species, do not have equal taxonomic status. The families grouped below (that exclude those almost entirely dominated by azooxanthellate taxa) are those determined from morphology, except for the new family Coscinaraeidae Benzoni & Arrigoni, 2012 determined from a combination of morphological and molecular taxonomy, and the restoration of three monospecific families. Morphology-based families are compared with DNA phylogenies in ‘Phylogenetic trees’ below.

#### Well-defined families

Name alternatives from remote past history aside, there are no morphological taxonomic issues with the following families: Acroporidae Verrill, 1902; Agathiphylliidae Vaughan & Wells, 1943; Coscinaraeidae Benzoni & Arrigoni, 2012; Dendrophylliidae Gray, 1847; Euphylliidae Milne Edwards, 1857; Fungiidae Dana, 1846; Merulinidae Verrill, 1866; Oculinidae Gray, 1847; Oulastreidae Vaughan, 1919; Pocilloporidae Gray, 1842; Rhizangiidae d'Orbigny, 1851; and Trachyphylliidae Verrill, 1901. Agathiphylliidae Vaughan & Wells, 1943 (the family of *Diploastrea heliopora*), Oulastreidae Vaughan, 1919 (the family of *Oulastrea crispata*), and Trachyphylliidae Verrill, 1901 (the family of *Trachyphyllia geoffroyi*) are monospecific families (J. E. N. Veron, unpubl. data). These families are all distinctive and, morphologically, the multispecies families listed above appear to be monophyletic.

#### Potentially divisible families

The following families have a genus or a group of genera that are traditionally included in the family with doubt: Agariciidae Gray, 1847; Astrocoeniidae Koby, 1890; Meandrinidae Gray, 1847; Pectiniidae Vaughan & Wells, 1943; Poritidae Gray, 1842; and Siderastreidae Vaughan & Wells, 1943. Of these, the inclusion of *Alveopora* in the Poritidae is of particular interest because *Alveopora* species have greatly reduced skeletal development, so much so that their inclusion in the Scleractinia at all was once a subject of debate (Bernard, 1903). Perhaps a minor matter, all *Alveopora* have 12 tentacles, unlike its nearest genus, *Goniopora*, which have 24. Although these genera are otherwise similar it is possible that this similarity is the result of convergent evolution. The Agariciidae currently contain two doubtful genera, *Coeloseris* and *Gardineroseris*. Additionally, the inclusion of *Psammocora* (formerly in family Thamnasteriidae Vaughan & Wells, 1943) in the Siderastreidae (by Veron & Pichon, 1976) is questionable. *Dichocoenia* does not clearly belong to the Meandrinidae. *Echinomorpha* and *Pectinia* are unlike each other, and also differ from the other genera of the Pectiniidae. *Stephanocoenia* does not clearly fit within the Astrocoeniidae. Morphologically, these families may be monophyletic as they stand or may only be monophyletic with specific deletions. It is noteworthy that of the genera noted in this group, five are monospecific and are candidates for their own monospecific families.

#### Over-extended families

The Faviidae Gregory, 1900 and Mussidae Ortmann, 1890 are large related families that have cores of closely related genera. These would make them broadly cohesive were it not for the presence of doubtful inclusions, especially *Cladocora*, *Parasimplastrea*, *Solenastrea*, and *Moseleya* in the Faviidae, and *Blastomussa*, *Micromussa*, *Acanthastrea*, and *Mussismilia* in the Mussidae. *Micromussa* and some species of *Acanthastrea* are so faviid-like that they are only included in the Mussidae on questionable development of mussid-like septa dentations and fleshy soft tissues. Morphologically, these families could be monophyletic as they stand or monophyletic with the aforementioned genera excluded; however, even with these exclusions, the Faviidae would remain very polymorphic. The inclusion of *Heterocyathus* in the Caryophylliidae Gray, 1847 follows tradition. This is a very large azooxanthellate family that clearly contains a wide spectrum of unrelated genera.

### Family trees

**Figure 4:**
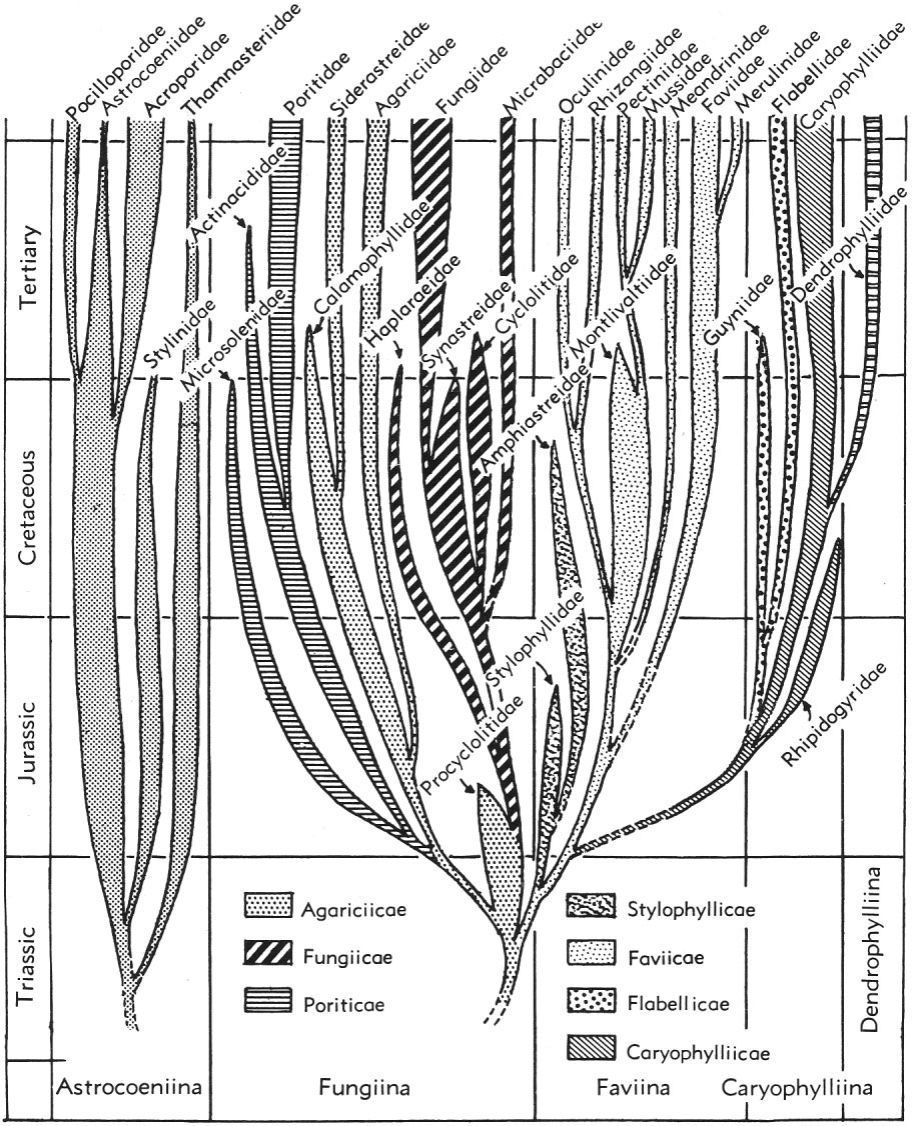
The family tree of Scleractinia (Wells, 1956).

**Figure 5:**
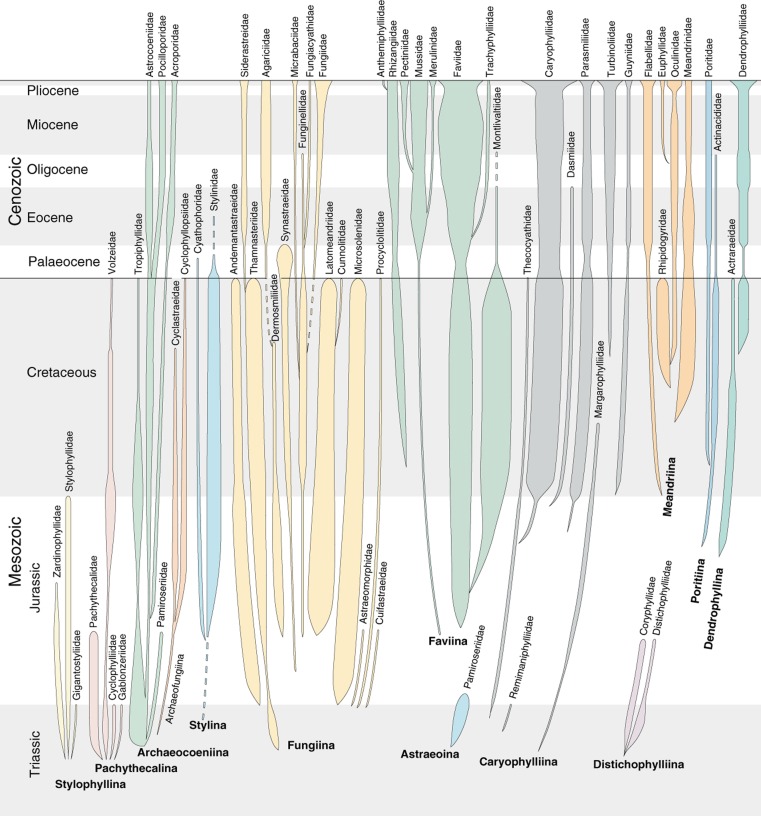
The family tree of Scleractinia (Veron, 1995, 2000a).

Three family trees have been published: those of Wells (1956; see Fig. 4), Roniewicz & Morycowa (60), and Veron (1995) (revised in Veron, 2000a; Fig. 5). All are based on skeletal structure incorporating the taxonomy of modern corals and an interpretation of the fossil record of the time. The tree of Roniewicz & Morycowa, reviewed by Stolarski & Roniewicz (2001), is largely derived from the skeletal microstructure of fossils as seen in thin sections, whereas the revision of Veron (2000a) incorporated the results of a molecular study (Veron *et al*., 1996). In brief, there is little agreement between the tree of Roniewicz & Morycowa (60) and the other two trees, with differences arising from the reliance on the methodology of thin sections and the focus on fossils. Differences between Wells (1956) and Veron (1995, 2000a) reflect the state of knowledge of the fossil record of these widely different times, taxonomic revisions made during this interval, and Wells' belief that the Scleractinia originated in the Middle Triassic as two separate clades: one being the Suborder Astrocoeniia Vaughan & Wells, 1943; the other being all other families. The only change between the original and revised trees of Veron is in the position of the Merulinidae, made in accordance with the aforementioned molecular study.

In evaluating any family tree it is important to note that all genera must be included. If, for example, family Mussidae was represented only by *Acanthastrea* in one tree and only by *Symphyllia* in another, the resulting two trees would indicate very different affinities with the family Faviidae. The same applies to extinct families. In principle, family trees depend on the comprehensive coverage of all component taxa. This requirement decreases the value of all family trees; however, the final elucidation of the phylogeny of extant Scleractinia is now exclusively in the realm of molecular studies.

### Molecular technology

Molecular studies made an uncertain start in the late 1980s using immunology and electrophoresis. This was accompanied by a protracted search for suitable primers (at James Cook University, Australia, and at the University of Hawaii) that worked for coral DNA. The first of these schools (using nuclear *28S* ribosomal DNA) was used to further investigate the family tree of Veron (1995), as mentioned above; the second (using *16S* mitochondrial DNA) resulted in the phylogenetic tree of Romano & Palumbi (1996). Both trees supported the integrity of the traditional families. Since then, differences between phylogenies indicated by morphology and molecular tools have been highlighted, even dramatized. This raises some general issues.

1. The primary focus of most morphologically based coral taxonomy is the species level. Genera are brought into question, or not, depending on comprehensive species-level comparisons and because all species must be assigned to a genus (see ‘Categories of species’ above). The family level is primarily used to group genera into a meaningful order for publication (see ‘Categories of genera’ above). This is a bottom-up process. The results of molecular phylogeny are generally observed top–down, independently of comprehensiveness. Thus it is hardly surprising that the two do not intermesh without conflict; however, the level of conflict (with taxonomy as opposed to phylogeny) is commonly overstated, as molecular studies tend to extend morphological results rather than contradict them.
2. The skill set of morphological taxonomists is centred on coral biology, skeletal architecture, and the taxonomic literature. The skill set of molecular biologists is centred on molecular technology. With several notable exceptions (see ‘Molecular taxonomic tools’ below), these widely differing skills are often not adequately combined, leading to errors in the identification of material collected for molecular studies. This issue is most prevalent in the Faviidae, where mistakes are common even at the generic level. Significantly, many species require both field and laboratory study for definitive identification (see ‘Species *in situ’* above) something that, so far, species selected for phylogenetic studies seldom get. Equally importantly, depositing voucher specimens in museums is an essential part of all taxonomy, but an opt-out if used a substitute for solid original identification.
3. There is a significant range of conflicting results among different publications that cannot be attributed to sampling error, but may result from different methodologies and different sectors of the genome being studied. Most studies try to address this by using both nuclear and mitochondrial DNA; however, some results are so conflicting that they span the deepest division within the Scleractinia, a clear indication that phylogenetic studies have a long way to go (see below).
4. The results of molecular studies are usually submitted to GenBank and are then retrievable via an accession number. There is no control of data quality in this process, thus allowing past errors stemming from methodology or sampling to exist in perpetuity, and to become widespread among those who use these data. For the molecular biologist, using archived data from other studies is a normal way of comparing new results with old, or for extending the comprehensiveness of new data; however, for the morphological taxonomist, seeing the resulting compilations can look like a new verification of what they believed to be an old mistake.
5. As with most science, career and funding opportunities are enhanced by results that appear new or different, prompting attention-seeking titles and also publications that are clearly premature.

### Phylogenetic trees

Despite periodic confusion, there are basic differences between a family tree intended to illustrate the evolutionary history of the Scleractinia at the family level and a phylogenetic tree, which is the result of a specific molecular study. Although both aim to illustrate phylogeny, the latter is entirely created from living tissue and is usually restricted to a particular group of taxa. The phylogenies of Veron *et al*. (1996) and Romano & Palumbi (1996) both indicate a deep division in the Scleractinia, which the latter authors nicknamed (somewhat inappropriately) ‘robust’ and ‘complex’ clades. At first sight these clades seem analogous to the two groupings of Wells; however, the mix of families involved is completely different.

Since that time many relevant studies have been published or attempted, culminating in the penetrating study of Fukami *et al*. (2008) using both nuclear and mitochondrial DNA from 127 species, 75 genera, and 17 families. They concluded that the majority of taxa at suborder and family levels are not monophyletic, which is hardly surprising given the ranges of categories of the families, genera, and species involved (see above); however, this motivated many further studies addressing the issue. In this process, the family divisions of Veron (2000a), which are largely based on Vaughan & Wells (1943) and Wells (1956), are usually referred to as ‘traditional’, whereas the families derived from phylogenetic studies are commonly called ‘revolutionary’ or ‘the new order’.

Of the three categories of families noted above, Fukami *et al*. affirm the doubts of all families listed in categories ‘2’ and ‘3’. Of category-‘1’ families, only the Dendrophylliidae remained unchanged; the Euphylliidae and Oculinidae were polyphyletic, but the study appears to affirm the monophyletic status of core members of the other families. Nevertheless, their data include some extraordinary observations: (1) that *Galaxea* is not in the Oculinidae but in the Euphylliidae; (2) that *Ctenella* and one species of *Pachyseris* are also in the Euphylliidae; (3) that *Cladocora* and *Solenastrea* are not in the Faviidae, but in the Oculinidae; (4) that *Pectinia* and *Mycedium* are not in the Pectiniidae, but in the Faviidae; (5) that *Oxypora* and *Echinophyllia* are also not in the Pectiniidae, but in the Mussidae; (6) that *Leptastrea*, *Psammocora*, *Coscinaraea*, and *Oulastrea* are all in the Fungiidae; (7) that *Alveopora* is not in the Poritidae, but the Acroporidae; and (8) that *Physogyra* is not related to *Plerogyra*.

Fukami *et al*. (2008) conclude that morphological characters ‘must be plagued by convergence’. With reference to details of the genera involved, the present author concludes that families of Scleractinia have not yet been well established by molecular methods (see ‘Ockham's razor’ below); however, the aforementioned ‘extraordinary’ results are not dismissible as mistakes of unknown origin, as most results of Fukami *et al*.'s study accord with generic-level morphological taxonomy, and some of the most unlikely molecular results have independent support (for example, in Kitahara *et al*., 2010). Independent support also extends to species level: for example, Benzoni *et al*. (2007), ahead of Fukami *et al*.'s paper, flagged affiliations of *Psammocora explanulata* Van der Horst, 1922 and *Coscinaraea wellsi* Veron & Pichon, 1976 with the Fungiidae, and more recently her group have placed both species not just in the Fungiidae as new genera, but specifically in the genus *Cycloseris* (Benzoni *et al*., 2012).

### Ockham's razor

Perhaps fuelled by the results of Fukami *et al*. 's (2008) study and subsequent updates of it, Budd, Fukami, Smith, and Knowlton have undertaken to ‘formally revise the classification of Scleractinia assigned to the suborder Faviina Vaughan & Wells, 1943 ’, of which the first part, family Mussidae, has currently been published (Budd *et al*., 2012). This study essentially aims to combine the phylogenies of Fukami *et al*. (2008) with Budd's work on the microcrystalline structure of Neogene Faviina. Although this is a seemingly unlikely combination, ‘formal’ revisions (historically meaning revisions without discussion) by geologists have precedents: T. W. Vaughan and John Wells were both geologists who created the definitive taxonomic catalogues of their time, primarily for fossil taxa. Later, Jean-Pierre Chevalier and Louise Beauvais, also geologists, did something similar (Chevalier & Beauvais, 1987); however, the ambit claim of a ‘formal revision’ warrants consideration, especially as the internal microstructure of skeletal elements does not define any family, genus, or species used in the taxonomy of extant corals (see ‘Fossils, taphonomy, and microcrystalline structure’ above). A first observation is that extensive name changing and the creation of new names (foreshadowed by Fukami *et al*., 2008) has been used in place of an argued revision of existing names (an issue referred to in ‘Type species’ above), in a process that enhances the visibility of Caribbean taxa (initiated by Fukami *et al*., 2004), which Budd *et al*. believe is essential for biodiversity and conservation studies.

Although such a ‘revision’ is likely to be different if based on Red Sea corals and their Tethyan ancestors, Budd *et al*.'s observations are of interest because they link thin sections, the primary methodology of generic-level fossil taxonomy, with extant corals. However, their publication is not about fossils, nor only about families and genera: it extends to species, not because species are the necessary entry point of molecular data, but as an intended species-level taxonomic revision. In so doing the authors adopted the species coverage of Veron (2000a) and ‘revise’ it through a library of historical generic designations (see ‘Historic collections’ and ‘Type specimens’ above), type-species issues (see ‘Type species’ above), and ICZN opinions (see discussion in ‘International Commission of Zoological Nomenclature’ above), and then made name changes to species reviewed via numerical taxonomy of morphometric data obtained from museum specimens (see ‘Fossils, taphonomy, and microcrystalline structure’ and ‘Morphometrics, cladistics, and pattern recognition’ above). This process provides many surprises. For example, the two Indo-Pacific species of *Scolymia*, *Scolymia australis* (Milne Edwards & Haime, 1849 (returned to an old genus *Homophyllia*) and *Scolymia vitiensis* (Brüggemann, 1877) (returned to an old genus *Parascolymia*), are placed in a new family along with *Moseleya*, *Micromussa*, and *Oxypora* (with Caribbean *Scolymia* having been placed in another family with other Caribbean mussids). For another curious Caribbean example, the moving of *Isophyllastrea rigida* (Dana, 1846) to the previously monospecific genus *Isophyllia* Milne Edwards & Haime, 1851 by Veron (2000a, referred to above) is retained, although Budd *et al*.'s molecular data indicate that this species and *Isophyllia sinuosa* (Ellis & Solander, 1786) are ‘identical’. Many questions arise as to the basis of such changes. For example, on what basis is *Montigyra kenti* Matthai, 1928 (known from a single specimen) classified with *Galaxea* Oken, 1815? And has the grouping of the Brazilian faviid *Favia leptophylla* Verrill, 1868 with the Brazilian mussid *Mussismilia* Ortmann, 1890 something to do with the proposed exclusion of Indo-Pacific *Favia* (re-named *Dipsastraea* de Blainville, 1830) from the Atlantic? And does microcrystalline structure support the inclusion of *Hydnophora* and *Caulastrea* in the same family, along with *Trachyphyllia*? What is most surprising of all is that these sorts of revisions have apparently been made without any original studies of living corals, except for the three species of the *Montastraea annularis* group. In effect this study attempts to link the morphological taxonomy and mindset of the pre-scuba era to molecular results, bypassing most of the intervening biological literature: a heroic leap indeed.

An alternative view is that DNA alone will ultimately determine the phylogeny of the Scleractinia. Certainly skeletogenesis needs to be studied in living corals (see ‘Fossils, taphonomy, and microcrystalline structure’ above) using both thin sections and scanning electron microscopy; however, this should be undertaken in tandem with other microstructural studies, especially of nematocysts and reproductive organs, in order to bring phylogenies determined by DNA into the realm of micromorphology. Once completed, the microstructure of skeletons can then be used to further enhance the fossil record, where structural details, including that seen in thin sections, are adequately preserved.

At this point in time it can only be observed that molecular and morphological phylogenies have not yet revealed basic conflict with the taxonomy of most existing species (where species have actually been studied, see below), but for others the differences that have arisen range from the unlikely to the apparently inexplicable. If there was a technical reason for the latter, experts in this field would have spotted it long ago, just as DNA contamination is easily detected. There are, however, potential explanations: perhaps the holobiont bacterial and viral soup that corals have always lived with could have been involved in the transfer of DNA between unrelated colonies at some point in their geological history. Alternatively, cross-fertilization might have occurred in corals at a remote time when surviving families were not as separate as they are now. For example, ultra-rare hybridization may once have occurred between a *Coscinaraea*-like coral and a *Cycloseris*-like coral, producing a surviving hybrid of unknown morphology but one that, through subsequent generations of introgression, retained the morphology of one of the parent species. The physical stage of such a process is easy to envisage: long-distance dispersal leading to extreme isolation is commonplace in corals, and introgression spanning geological intervals can clearly be driven by continental boundary currents capable of transporting the genes of one parent species whilst blocking any return pathway of the hybrid. This is a mechanism that might explain the inexplicable, however unlikely that explanation might initially appear to be. If whole-genome studies can resolve such speculation, a good starting point would be the two species of *Coscinaraea* (*Coscinaraea marshae* Wells, 1962 and *Coscinaraea mcneilli* Wells, 1962) now confined to southern Australia by boundary currents. In principal, this is an aspect of the driving mechanism of reticulate evolution described below.

Aside from such speculation, it should be noted that the same sorts of issues – the separation of molecular evolution from morphological evolution – arise in other major taxa, even in extensively studied vertebrates where morphological and molecular taxonomy are in basic conflict (Losos, Hillis & Greene, 2012).

The time will certainly come for a complete reappraisal of coral taxonomy from top to bottom, and, critically, this will be based on entire genome studies of all accessible species. Perhaps it might then be clear as to why *Alveopora* should be in the Acroporidae or why *Coscinaraea wellsi* should be *Cycloseris wellsi*. If such phylogenies become unarguable (which might involve the identification of dormant genes, an exceedingly difficult undertaking), then the identification of corals, which already has a reputation for being difficult for non-taxonomists, will take some interesting turns; however, in all cases the overriding need is to reveal operational taxonomic units that allow users of taxonomy to get on with their studies.

### Molecular taxonomic tools

A wide range of taxonomic questions can only be answered using molecular methods, and it is certain that the continuing proliferation of molecular studies will have a major impact on most aspects of coral taxonomy and biogeography. To date, molecular studies have been based on current morphological taxonomy, not for any scholarly reason, but for sampling purposes. These have yielded clades of many sorts that, at species level, go by various names, including ‘operational taxonomic units’, ‘evolutionarily significant units’, ‘morpho-groups’ and ‘cryptic species’, all of which raise many questions, such as ‘when is a species not a species’?, ‘what is the difference between a species and an ecomorph'? (see ‘Subspecies taxon units’ above), and ‘is there a clear difference between phenotypic plasticity and genotypic division'?

In principle, molecular taxonomy (as opposed to phylogeny) is set to go through three developmental phases.

1. Using molecular markers selected because they yield results (a ‘whatever works’ approach). All but the most recent studies to date are in this category, and curiously most rely partly or wholly on mitochondrial DNA. Mitochondrial DNA would not be expected to code for morphology, and would certainly not be expected to be more informative than nuclear DNA; however, the former frequently works when the latter does not, and these studies appear to be valid.
2. Revisions of this work using the entire genome. This technology, which is now available, should ultimately provide answers for most questions raised above, although there will be inevitable conflicts arising from the different evolutionary histories of different loci. This moves taxonomy into a field dominated more by information technology (to elucidate whole-genome structure) than by molecular technology.
3. A molecular taxonomy where samples are semi-randomly selected in the field by techniques such as barcoding rather than depending on specific field characters. This would bring the principles and predictions of reticulate evolution into sharp focus (see ‘The last frontier’ below).

**Figure 6:**
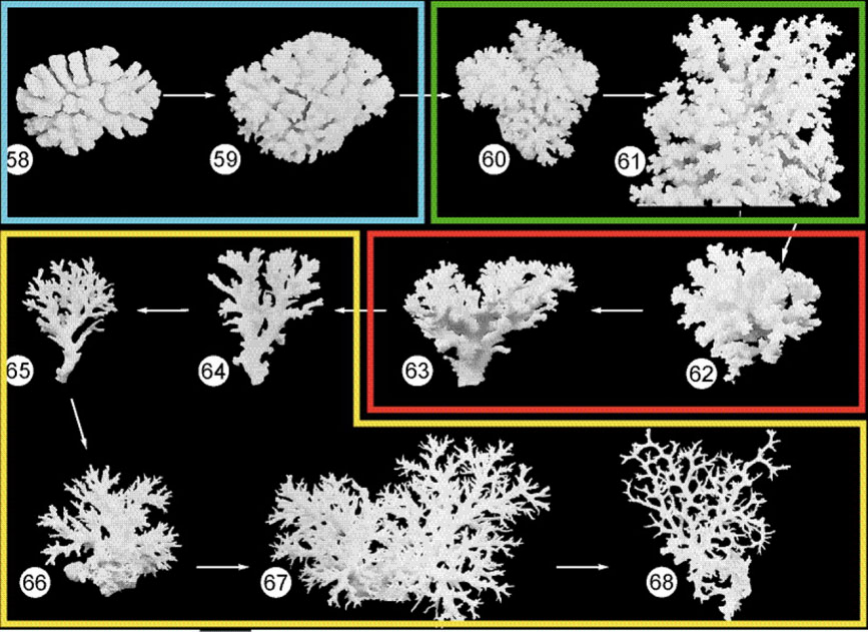
Morphological gradation in *Pocillopora damicornis* colonies as illustrated by Veron & Pichon (82), then re-classified by Schmidt-Roach *et al*. (2013b) using molecular methods. The latter study revealed that this compilation includes two cryptic species: *Pocillopora brevicornis* (the blue box) and *Pocillopora acuta* (the yellow box). These species are being re-described (Schmidt-Roach *et al*., in press). Numbers are figure numbers in the original publication.

Once again, *Pocillopora damicornis* can be used to illustrate progress and the present state of knowledge. In a detailed study of mitochondrial lineages, Schmidt-Roach *et al*. (2013b) found that *Pocillopora damicornis* inferred from Veron & Pichon's (82) original study (Fig. 6) forms a species complex, characterized by high levels of plasticity within clades and cryptic points of differentiation between clades. Two ecomorphs of Veron & Pichon (82) are ranked as distinct or possibly distinct species: *Pocillopora brevicornis* Lamarck, 1816 (the blue box, still questionable) and *Pocillopora acuta* Lamarck, 1816 (the yellow box) (Schmidt-Roach *et al*., 2013b). The red box represents aberrant *Pocillopora verrucosa* (Ellis & Solander, 1786), leaving only the green box as *Pocillopora damicornis*.

Significantly, Figure 6 is a compilation of morphologically unusual colonies (out of about 150 studied), so they do not quantitatively represent what is seen *in situ*. Schmidt-Roach *et al*. (in press) confirm that *Pocillopora damicornis* is a highly polymorphic species that is now reliably separated from *Pocillopora acuta*, except in colonies from very sheltered habitats such as mangrove roots. At the other extreme, *Pocillopora brevicornis* (the correct name of which has yet to be confirmed) appears to be restricted to upper reef slopes, where it may also be difficult to distinguish from *Pocillopora damicornis*. On a broader geographic scale, Schmidt-Roach *et al*. (in press) have shown that there is a latitudinal component to the morphological plasticity of *Pocillopora damicornis*, so that colonies from the highest latitude locations of both the east and west coasts of Australia have greater genetic similarity with each other than with their tropical counterparts. They also conclude that *Pocillopora damicornis* in the far-eastern Pacific is genetically more akin to *Pocillopora verrucosa* (although I found these colonies to be clearly morphologically *Pocillopora damicornis*) and, surprisingly, that Hawaiian *Pocillopora molokensis* Vaughan, 1907 is a probable synonym of *Pocillopora verrucosa*.

Almost certainly, cryptic species will also occur in association with *Pocillopora damicornis*-like assemblages in other countries, and similar associations will also occur with *Pocillopora verrucosa*-like assemblages and probably with other *Pocillopora* species as well. This naturally begs the question: ‘how many species of Scleractinia are mainstream and how many are cryptic or yet to be discovered?’ and ‘will coral identification become so complex that it will become the exclusive domain of specialist taxonomists?’

In an attempt to address these questions, the ‘Categories of species’ (listed under this subheading above), groups ‘d’, ‘e’ and ‘f’ (each indicating the likely presence of cryptic species), combined amount to 15% of all valid species (J. E. N. Veron, unpubl. data). If these mask an average of three cryptic species each, there would be about 970 species in total. Of course this number excludes species that are so rare that they have not yet been discovered, species that have been discovered but have unrecognizable descriptions, species that have been discovered but have not yet been described, and additional species that are likely to be revealed from molecular studies of geographic variation. This indicates that there are at least 1000 zooxanthellate Scleractinia worldwide that are sufficiently distinctive to be operational taxonomic units. If so, most species should remain identifiable from their morphology, although many will require a high level of expertise.

## The Last Frontier

This brief overview ends with concepts describing evolutionary mechanisms and biogeographic pattern formation, subjects that do not fit comfortably under the banner of ‘taxonomy’, but which nevertheless directly impinge on what species are taxonomically, and how they are distributed geographically.

### Reticulate evolution

The concept of reticulate evolution has been variously dubbed the same thing, more-or-less, as ‘introgression’, ‘hybridization’, ‘vicariance’, ‘anti-Darwinian heresy’, and ‘a statement of the obvious’. It is in fact all of these things in part but none in whole. Clearly, this concept has different meanings for different people, depending for the most part on their field of speciality.

Since well before Darwin, species have been regarded as the fundamental building blocks of nature, units that can be named, described, mapped, and studied. This is an enduring concept that certainly applies to corals, but with qualifications that significantly impinge on species-level taxonomy and biogeography.

**Figure 7:**
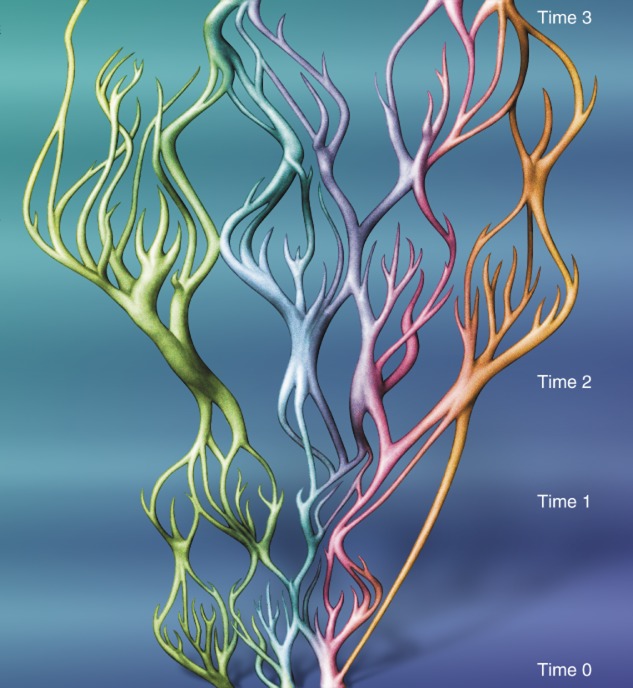
A hypothetical view of reticulate evolutionary change within a group of species belonging to a single syngameon, after Veron (2000a). At time 0 the group forms three principal species, each of which is distinct and widely dispersed by strong ocean currents. At time 1 the group forms many species that are geographically isolated because of weak ocean currents. At time 2 the group forms four species that are again widely dispersed by strong currents. Over the long geological interval to time 3 the group has been repackaged several times.

Figure 7 shows why reticulate evolution is sharply contrasted with the Darwinian view. In contrast to neo-Darwinism (effectively Darwinian evolution and genetics combined), which can be envisaged as an evolutionary tree producing ever-finer branches, this concept sees species as semi-arbitrary items of genetic continua rather than as units. These items of continua have no time nor place of origin, for they are being continually re-grouped within their syngameon (genetically isolated *groups* of potentially interbreeding species) in both space and time. Importantly, reticulate evolution is under physical environmental control, not biological control. Continual changes in ocean surface currents create continually changing patterns of larval dispersal, and consequently ever-changing patterns of genetic connectivity. Although Darwinian evolution would always occur simultaneously with reticulate evolution, the mechanisms are different. Darwinian evolution is driven by competition between species resulting in morphological changes through natural selection, whereas reticulate evolution is driven by ocean currents resulting in genetic changes via the making and breaking of genetic contact.

If the characteristics of both types of evolution are compared (Veron, 2000b), reticulate evolution may seem incompatible with neo-Darwinism, yet there is a point where the two concepts meet without conflict: the point involves the difference between a genetically isolated species and a syngameon. Species which *are* genetically isolated can evolve through Darwinian natural selection because they can remain genetically cohesive in space and time; however, those that are a part of a syngameon will not do likewise, because changes in the gene pool of a single species (through natural competition) will become diluted when combined (through ocean current transport) with the gene pools of other species. In effect, there is a similar relationship between a species and its parent syngameon as there is between a population and its parent species. Both are similarly affected by changing patterns of connectivity: changes dominated by rare events.

In taxonomy, a syngameon is an invisible taxon level that can only be detected, in any animal or plant, by breeding experiments or by the study of whole genomes of all component species. In corals that have been artificially hybridized (Willis *et al*., 1997, 2006), parent species can be very different from each other, and their progeny might be similar to one parent or to neither parent. Many further complications may arise with corals, including the possibility that some colonies are chimeras formed by the union of several original larvae, potentially of different species, although this is not an evolutionary mechanism.

The taxonomic relevance of this is that most species are probably part of a syngameon, which means that they are not stable genetic units even though they may be sufficiently distinguishable at the present point in time to form operational taxonomic units. Importantly, the more such species are studied the more intractable their taxonomic status will appear. When a species is part of a syngameon, the question ‘when is a species not a species’ has no clear answer, an issue that creates both morphological and genetic fuzziness, especially in species that have very large distribution ranges, and in those that have geographically isolated components.

### Reticulate pattern formation

The concept of reticulate evolution came to corals from biogeographic studies revealing that details of the characters of a species in one country may gradually change when the same species is studied in progressively more distant countries (Veron, 1995). This sometimes necessitates an arbitrary decision as to what the species is and where its distribution boundaries are. For example, if a supposed species in the Red Sea has slightly different characters from the same supposed species on the Great Barrier Reef, the species may be regarded as a single species if these differences intergrade geographically. If, however, the two overlap and are still clearly distinguishable, perhaps in the Coral Triangle, they can confidently be regarded as separate species. At the present point of knowledge these sorts of decisions have been made at least initially in the taxonomy of most species. Patterns that now remain to be resolved are mostly more complex, involving interlinked parts of continua where points of variation *within* a single species are indistinguishable from points of variation *between* similar species. This creates an endless dilemma, for humans cannot easily communicate in terms of continua: they need discrete units of some form or other to do so. Nevertheless, recent studies have started to address this issue, currently with genetic evidence of the existence of a syngameon in a common group of *Acropora* (Ladner & Palumbi, 2012).

### Where molecular taxonomy and biogeography meet

At some future time when the genetic composition of what ‘species’ are is well understood, the genetic patterns of reticulate evolution predicts that there will be many more species than are currently recognized, but that most will *still* have fuzzy morphological, genetic, and geographic boundaries.

The massive literature on the subject ‘what are species?’ does not appear to have achieved a clear resolution for any major taxon, presumably for the simple reason that none exists; however, such theoretical considerations do not diminish the value of species names in current use, provided that all are accepted as concepts, concepts that have changed in the past and will continue to change in the future. The name *Pocillopora damicornis* was originally a very vague concept; it was made less vague by *in situ* studies, and has now become further refined by molecular and *in situ* studies combined. The name *Porites lobata* was also an ill-defined concept, and as yet largely remains so.

Reticulate pattern formation (see above) predicts that a distribution map of, for example, *Pocillopora damicornis*, at some future time, is likely to include all locations where this species is currently recorded, as well as additional locations where other species of *Pocillopora* have been recorded. The map may look something like a barometric chart, with some places of high affinity (equivalent to high barometric pockets of the chart) and other places of low affinity (equivalent to low barometric pockets of the chart), separated by patterns of intermediate affinity (the isobars of the chart). Perhaps *Pocillopora damicornis* will have the Coral Triangle and other places downstream from it as areas of highest affinity (if the molecular neotype is from that region), and perhaps the Far Eastern Pacific will be an area of lowest affinity if the *Pocillopora damicornis*-like corals there are confirmed to be more akin to *Pocillopora verrucosa*.

In a similar vein, *Porites lobata*, with its type locality in Fiji, is likely to have a very different future distribution map, as suggested by the fuzziness of its current morphological and distribution boundaries. This species may have its present Indo-Pacific-wide distribution confirmed, or perhaps it will be broken-up by cryptic species, as is illustrated in the cycle of divergence in Figure 7.

There are many issues for conservation in these future biogeographic patterns, especially for remote regions that are likely to have higher levels of endemism than are currently realized. Whether this is so or not, corals are likely to remain good indicators of reef diversity and of broad-scale patterns, so that the global prominence of the Coral Triangle region (Veron *et al*., 2009) is unlikely to change.

## Mapping a Future Pathway

In the next instant of geological time, less than a century of ours, Scleractinia may be facing a level of devastation as great as any in their past existence. Are coral taxonomists going to see it as their role to debate the name of the last coral standing? Of course by then such debate will be irrelevant, but between now and then there are choices. Some aspects of coral taxonomy, especially molecular phylogenetics, are mostly standalone endeavours; however, species-level taxonomy is not. Species names are important because they are what links information of any kind to that species. To make the point, if names were removed from all coral publications we would be left with hundreds of thousands of independent items of information, which would be meaningless. It follows that if we have two or more names for the same species then our knowledge of that species will eventually become divided two or more times (except for those taxonomists who follow such histories).

The advent of molecular taxonomy has challenged morphological taxonomy on many fronts, although, contrary to the claims of some, it is highly supportive of concepts and outcomes derived from *in situ*-based morphological taxonomy and biogeography, providing welcome tools to take these concepts to a higher level. Some authors (Benzoni *et al*., 2007, 2010; Forsman & Birkeland, 2009; Pichon, Chuang & Chen 2012; Schmidt-Roach *et al*., 2013a, to name a few) have elegantly combined both fields to produce thoughtful and progressive outcomes: taxonomy that will stand the test of time. Others appear to have scant knowledge of what they originally collected, or of any need to retain nomenclatorial stability, or of any broader context for their results.

Be that as it may, all taxonomy has its nomenclature resting on the same historical foundation, one overshadowed by supposition about type specimens and therefore prone to failure at any challenge.

The ICZN once appeared to embrace the concept that name changes were not acceptable if they increased confusion, and indeed this may still be the case; however, there is nothing to *prevent* taxonomists from making such changes. Many of the issues stemming from a bygone age described in this overview *can* be curtailed, but most are not. This article argues that coral taxonomy will avoid looming pitfalls if: (1) well-established names are retained, unless there are compelling reasons to change them; (2) nomenclatorial priority is not allowed to be a reason for changing a well-established name; (3) names of fossils are not used for extant species (excluding rare instances where the holotype is unambiguous); (4) rules of Latin declension are not given priority over the needs of name stability and information technology; (5) the names of well-established genera are not subject to change because of taxonomic issues with type species; (6) when the identity of a type specimen of a well-established species is deemed inadequate (as so many are), it is replaced with another specimen that does the job; and (7) a mechanism is devised that allows new type specimens with preserved soft tissue to share equal status with older skeletal holotypes. Some of these reforms seem straightforward; others would involve substantial changes to existing ICZN rules, others have procedural difficulties, especially those where consensus is called for; however, if such reforms were implemented, ‘the tyranny of the past’ would no longer exist. The alternative, the present *status quo*, will keep coral taxonomy permanently mired in its historical past.

In conclusion, the way ahead cannot now rest on any single publication, methodology, or concept; it must rest on open-access, updatable websites that link taxonomic, phylogenetic, biogeographic, ecological, palaeontological, environmental, and bibliographic data. Such information sources allow taxonomists of all persuasion to see the results of their endeavours in a broad context, and not just within the confines of their own subdiscipline. Some existing websites undertake this task within their designated field. Another called ‘Corals of the World’, which underpins this overview, is due for release in 2014. Access to information that websites can uniquely provide give hope that coral taxonomy can remain integrated and, most importantly of all, relevant to the enormous challenges that lie ahead.
